# Optimization of MoNiCr Alloy Production Through Additive Manufacturing

**DOI:** 10.3390/ma18010042

**Published:** 2024-12-26

**Authors:** Michal Duchek, Daniela Nachazelova, Martina Koukolikova, Michal Brazda, Pavel Ludvik, Josef Strejcius, Zbysek Novy

**Affiliations:** 1 COMTES FHT a.s., Prumyslova 995, 334 41 Dobrany, Czech Republic; 2 Centrum Vyzkumu Rez s.r.o., Hlavni 130, 250 68 Husinec, Czech Republic

**Keywords:** nickel alloy MoNiCr, additive manufacturing (AM), directed energy deposition (DED-LB), miniature tensile test specimens (MTTs), solidification cracking, molten salt reactor (MSR)

## Abstract

One of the concepts behind Generation IV reactors is a molten salt coolant system, where the materials for the reactor itself and for the primary and secondary circuit components are subjected to extreme chemical and thermal stresses. Due to the unavailability of these materials, a nickel–molybdenum alloy known as MoNiCr has been developed in the Czech Republic. This paper discusses the manufacturing process for the MoNiCr alloy, covering conventional casting technology, forming, powder atomization, additive manufacturing (AM) using the directed energy deposition (DED-LB) process, and final heat treatment. Special attention was given to the quality of the input powders for additive manufacturing, particularly regarding the optimization of the chemical composition, which significantly influenced the quality of the additively manufactured components. AM enables the realization of complex structural designs that are critical for energy applications, despite the high susceptibility of the MoNiCr alloy to solidification cracking. Through AM, a test body was successfully produced with a maximum defect rate of 0.03% and the following mechanical properties: a yield strength (YS) of 279 MPa, an ultimate tensile strength (UTS) of 602 MPa, and an elongation (El) of 51%.

## 1. Introduction

Modern nuclear reactors have consistently been a major source of low-carbon energy. Nevertheless, the next generation of reactors is being developed to ensure better efficiency, sustainability, and safety. Molten salt reactors (MSRs) are one of the most promising advanced Generation IV reactor systems. This reactor system is currently experiencing increased interest from organizations and companies that are also designing small modular reactors (SMRs) [1].

Current research and development is now mainly focused on the level of basic theoretical studies on the MSR system, complemented by experimental testing of selected parts of this technology. In the area of materials research, this mainly involves testing the technological, corrosive, and thermomechanical properties of selected structural materials that could be used for the construction of an MSR. A nickel–molybdenum-based alloy with the commercial name Hastelloy N was developed for a reactor system cooled by the fluoride melt LiF-BeF_2_ in the 1960s [2]. After 2000, when interest in MSR development was renewed, Hastelloy N was not commercially available, and this led to the development and testing of other (alternative) commercially available nickel materials that could be used for MSR design. Typical representatives of these newly developed alloys are the Czech alloy MONICR, the Russian alloy HN80M, the French alloys EM-721 and EM-722, and the Chinese alloy GH3535 [3].

The molten salt corrosion performance of these alloys depends not only on their chemical composition but also on their microstructural characteristics. Findings have indicated indicate that the MoNiCr alloy is the least affected by exposure to FLiNaK molten salt when compared to GH3535 and Hastelloy N. This superior performance is attributed to its chemical composition, high-angle grain boundary length (grain size), and the absence of large (M6C) carbides [1]. Corrosion test results for seven different construction materials (stainless steel AISI 316L; Incoloy 800HT; Inconel 600; Inconel 625; MoNiCr; and HN80MTY) in molten NaF-NaBF_4_ salt at temperatures of 550 °C or 700 °C were published in [4]. The purposefully developed molybdenum–nickel-based superalloys MoNiCr and HN80MTY show the best corrosion-resistance performance among the tested construction materials, which makes them the recommended construction materials for future MSRs from the pool of tested materials. The results of tests conducted at the Kurchatov Institute demonstrated good compatibility between nickel–molybdenum alloys and metal fluoride molten salts containing the fuel additives UF4 and PUF3 at temperatures reaching up to 750 °C [5]. This study considers HN80MTY as the material with the highest resistance to intercrystalline tellurium cracking in fluoride molten salt.

In comparison with data on corrosion resistance, only minimal information is available in the literature on the mechanical and technological properties of nickel–molybdenum alloys suitable for molten salt environments. The presented experimental program deals with a MoNiCr alloy as a promising material for such intensive corrosion attacks. Namely, its metallurgical and additive processing will be described, and the resulting mechanical properties and microstructure features will be compared. MoNiCr, after proper processing, is a single-phase superalloy with an f.c.c. solid-solution microstructure.

The metallurgical processing of MoNiCr alloys should respect specific technological features concerning the atmospheric pressure, batch material purity, the order of melting of individual components, gas homogenization during melting, etc. Special attention must be paid to the method of alloying and to multi-stage deoxidation. In addition to deoxidation, sulfur elimination seems to be a critical point during melting. At the same time, it is necessary to optimize the content of accompanying elements to improve the deoxidation process and to achieve the desired mechanical properties [6].

In terms of the forming process, the key steps seem to be the transition of the cast state of the material to the state of a formed recrystallized microstructure with homogenous fine grains [7]. Nickel alloys can withstand a significantly higher deformation level if compressive stress prevails during their forming. The forming process, which mainly induces compressive components of stress, increases the probability that the material will achieve the required level of deformation without cracking [8]. In the formation of MoNiCr components, recrystallization plays a critical role in ensuring successful processing. This process runs very slowly or may not occur at all if unsuitable conditions are applied. Appropriate ranges of the temperature and strain rate have been identified, and furthermore, the impact of cold deformation on the progress of recrystallization has been determined [9,10].

Additive manufacturing (AM), commonly referred to as 3D printing, has emerged as a revolutionary process [11]. With AM, the production process becomes significantly simpler, as it facilitates the combination of different materials in powdered form and allows for adjustments to material compositions as needed. Recent advancements in AM processes have facilitated the fabrication of complex geometries of multi-material components with optimized mechanical properties [12,13,14]. The choice of AM processes depends on the material system, the desired properties, and the specific application requirements.

The directed energy deposition (DED-LB) of metals represents a family of additive manufacturing processes employed to create the final designs of samples for testing. It utilizes a directed heat source to melt feedstock material. The DED-LB of metals facilitates the additive manufacturing of large-scale metallic components with significantly higher deposition rates compared to other AM processes, such as powder bed fusion (PBF-LB). Generally, components produced using the DED-LB process are characterized by their large part size and relatively lower resolution when compared to PBF-LB [15]. This process is employed not only for part fabrication but also for surface cladding and part restoration. Notable applications include hip implants, blisks, large-scale aerospace components, and conformal cooling channels, among others [15]. One of the most remarkable capabilities of DED-LB is the fabrication of Functionally Graded Materials (FGMs), which exhibit superior mechanical properties [16]. Moreover, the ability to process nickel-based superalloys [17] with relative ease makes DED-LB particularly suitable for applications in high-temperature and corrosive environments, further expanding its utility. This specific area of research is further developed in the presented manuscript. The additive manufacturing of MoNiCr alloys opens the possibility of producing parts with significantly more complex shapes compared to conventional processes. The lack of data describing the relations between the chemical composition, technological procedure, and resulting properties requires further research into this alloy preparation process. 

However, a common issue in nickel-based superalloys, especially during additive manufacturing processes, is their susceptibility to solidification cracking [12]. This study aims to address this challenge by adjusting the chemical composition and refining manufacturing techniques to minimize defect formation, enhance material cohesion, and achieve a balance of the mechanical properties required for high-performance applications. Solidification cracking represents a significant metallurgical challenge in nickel-based alloys, often affecting the integrity and performance of components manufactured using processes involving rapid cooling, such as welding and additive manufacturing. This phenomenon arises because, as the material cools, the last portions of liquid solidify at grain boundaries, where shrinkage and localized stresses create weak points [18]. This can lead to deformation shifts at the grain boundaries, resulting in voids and cracks. Understanding and controlling solidification behavior is, therefore, crucial for improving the structural integrity of nickel-based alloys in high-temperature applications [12,19].

The quality of powder plays a critical role in additive manufacturing [20], especially in metal 3D printing. Powder properties, including particle shape, size distribution, bulk density, and flowability, directly impact the quality of the final part. Spherical particles with a narrow size distribution ensure better flowability and even distribution during the printing process, leading in higher density and improved mechanical properties of the final product. Therefore, careful selection and quality control of the powder are essential to achieve optimal results in additive manufacturing [21,22].

This research focuses on the development and optimization of the MoNiCr alloy for additive manufacturing applications, primarily aimed at advanced energy systems such as Generation IV molten salt reactors. The optimization of chemical composition, mainly DED-LB process parameters, and resulting mechanical properties, supported by metallography analysis, will be the most important results of the presented research.

## 2. Materials and Methods

### 2.1. Preparation of MoNiCr Alloy for Additive Manufacturing

The aim of the research was to develop and validate the nickel alloy MoNiCr for applications in additive manufacturing. The experiment focused on controlling process parameters and material properties to ensure that the resulting material achieved the required chemical composition and purity, which is crucial for its further use in demanding operating conditions.

A key element was the preparation of the alloy using a vacuum induction furnace capable of melting under both vacuum and protective inert gas, preventing contamination of the melt by the surrounding atmosphere and achieving high material purity. The furnace accommodates crucibles with capacities of 50 and 500 kg, with ingots for this work weighing 50 kg. During the alloy development, the chemical composition was optimized to improve corrosion resistance and enhance material properties for forming processes. Adjustments were made to the levels of Mn, Si, Al, and Ti, ranging from 0.001 to 0.25%, with some alloying strategies eventually omitting these elements entirely. To monitor the chemical composition, an optical emission spectrometer Q8 Magelan (Bruker-Elemental, Kalkar, Germany) was used, allowing real-time observation of the chemical composition during melting.

As part of the compositional adjustment, trace elements in the raw materials were reduced to improve the metallurgical quality of the molten metal. These measures resulted in a reduction in dissolved gases (O, N) in the alloy to levels slightly above 10 ppm, as measured by the G8 Galileo gas analyzer (Bruker-Elemental, Kalkar, Germany). Additional refining processes were tested, utilizing slag with a high Ca content and the passage of inert gas (Ar) through the melt before deoxidation. These processes aimed to further enhance the metallurgical quality and purity of the alloy.

The chemical composition of the MoNiCr alloys used for additive manufacturing is provided in Table 1 and Table 2.

The ingots were further processed to obtain a suitable preform for powder atomization. Hot forging was performed using a hydraulic forging press with a maximum forging force of 2500 tons. This equipment allows for both die and open-die forging. Chamber furnaces without protective atmospheres were used to heat the material. Rolling of the MoNiCr alloy was carried out on a reversing rolling mill, enabling both hot rolling (two-high roll arrangement) and cold rolling (four-high roll arrangement), with a rolling force of 600 tons and rolling motor torque of 300 kNm. Cold rapid forging was performed using a rapid radial forging machine to produce the final rod preform.

This was followed by ultrasonic atomization. The rod was fed under a plasma column, where the material melted onto the atomization platform. The melt was sprayed through platform oscillation in an inert argon atmosphere (Ar 5N9). The process was carried out under 10 ppm O_2_ in the chamber. A comprehensive powder analysis was conducted using a HORIBA LA-960 V2 (HORIBA Ltd., Kyoto, Japan) particle size distribution analyzer, following the ISO 13320 standard [23]. This analysis utilized laser scattering to accurately measure particle size and determine particle fraction at specified sizes. The powder’s flowability was evaluated through testing in compliance with ASTM B213 standards [24]. This test provides a measure of the ease with which the powder flows, a critical parameter in additive manufacturing, as it influences layer uniformity and consistency during deposition. Ensuring optimal flowability is essential for achieving high-quality builds and minimizing potential defects caused by irregular powder distribution.

A detailed analysis of the powders used for additive manufacturing and the specific printing parameters are described in Section 3.

### 2.2. Additive Manufacturing

**Figure 1:**
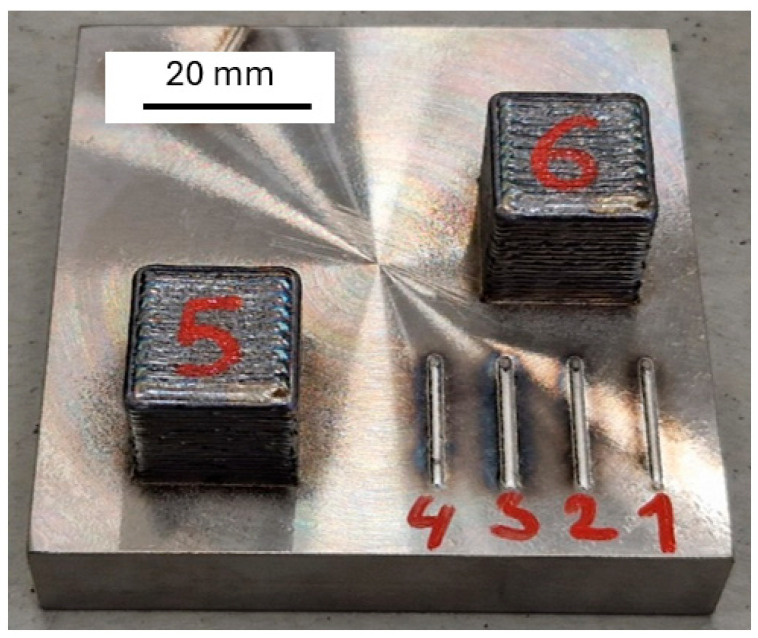
Deposited samples of 2-MoNiCr alloy.

The nickel alloys 1-MoNiCr and 2-MoNiCr were, after atomization, additively manufactured. The material blocks were produced by the DED-LB of metals using an INSSTEK MX-600 system (InssTek, Daejeon, Republic of Korea), equipped with a 2 kW yttrium fiber laser, operating in a protective argon 5.0 atmosphere. The printing parameters are listed in Table 3 and Table 4. The machine uses a 5-axis printing system without supports and with a total building space of 450 × 600 × 350 mm. The main advantage of the INSSTEK MX-600 system is the direct metal tooling (DMT) system used, which monitors the printing process using two cameras and adjusts the laser power during printing to maintain a stable layer thickness. The dimensions of the blocks were 20 × 20 × 20 mm^3^. Two printing modules, SDM800 and SDM1600, were used by additive manufacturing of the 1-MoNiCr alloy, while only the SDM1600 module was used for the fabrication of the 2-MoNiCr alloy samples. An example of the deposited samples of the 2-MoNiCr alloy, produced using the SDM1600 module, illustrates the general appearance of the alloy post-deposition (Figure 1). The samples were cooled in air after deposition. After additive manufacturing, part of the 2-MoNiCr alloy samples were heat-treated, specifically by solution annealing in a CLASIC vacuum furnace. The CLASIC vacuum furnace is designed for high-precision thermal processing. It features internal dimensions of ø300 × 200 mm and operates at temperatures up to 1600 °C. The furnace supports a range of atmospheres, including argon, hydrogen, and vacuum, with a maximum vacuum level of 10^−3^ bar. The heating was conducted at a rate of 10 °C/min up to 1200 °C, followed by a one-hour temperature hold in a protective argon atmosphere. The samples were cooled in water.

Table 5 represents an overview of samples prepared as part of the presented experimental program, which were subsequently characterized.

It is important to note that several factors influence the quality of the DED-LB process of metals. For our equipment, the most critical aspect is the proper adjustment of the optical system, specifically aligning the laser beam with the center of the nozzles. Another significant input parameter is the quality of the powder. The powder particles must be spherical, and so-called satellites should not be present. The powder should meet the flowability requirements as per ASTM B213 [24]. The particle size distribution (PSD) curve should follow a Gaussian distribution.

### 2.3. Microstructure Characterization

The methods and techniques employed for the microstructural characterization of the MoNiCr alloy are outlined, emphasizing their importance in achieving the required mechanical properties. The metallographic sample preparation process included grinding and polishing according to standard procedures, with the final polishing step performed using a 0.25 μm OP-S Non-Dry colloidal silica suspension on a Tegramin 30 (Struers GmbH, Ballerup, Denmark). Observations of the samples’ microstructure were made using a Nikon Eclipse MA200 light microscope (LM) (Nikon, Tokyo, Japan) supported by NIS Elements 5.2 software for digital imaging and analysis (Nikon, Tokyo, Japan). For a more detailed microstructural analysis, a JEOL IT 500 HR scanning electron microscope (SEM) (JEOL Ltd., Tokyo, Japan) equipped with an Octane Elite Super EDS analyzer (EDAX LLC, Mahwah, NJ, USA) was utilized. Data collection, analysis, and post-processing were carried out using TEAM 4.5 software (EDAX LLC, Mahwah, NJ, USA).

### 2.4. Mechanical Properties

The local mechanical properties of the material were assessed using miniaturized tensile specimens, a widely adopted approach in the additive manufacturing (AM) field, as documented in numerous studies [17,25,26].

**Figure 2:**
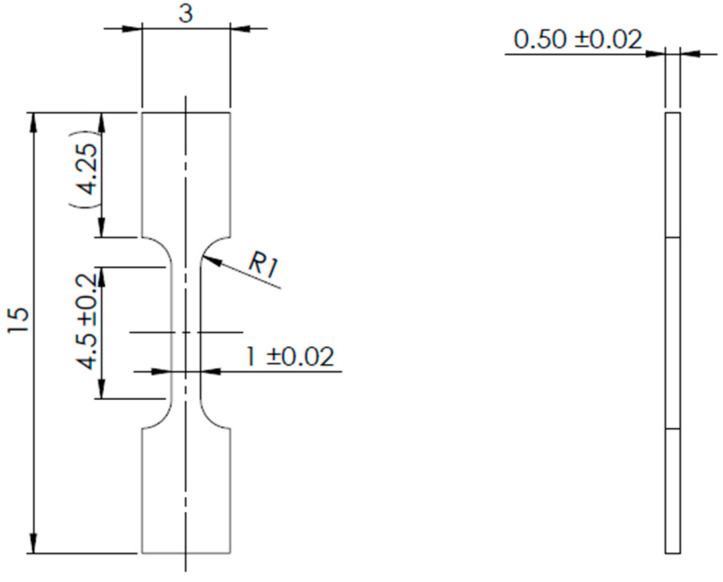
Geometry of miniaturized tensile test specimen.

Three test specimens (Figure 2) were prepared from each block using a wire electrical discharge machine (WEDM) and tested in compliance with ISO 6892-1 [27]/ASTM E8 [28] standards. The tests were performed on a TiraTest universal testing machine equipped with a 10 kN load cell under quasi-static conditions at room temperature, with a strain rate of 0.00025 s^−1^ (A222). The initial dimensions of the specimens were accurately measured using a micrometer, followed by the application of a stochastic black-and-white pattern to enable deformation tracking via an optical system. Given the small specimen size, a non-contact Mercury RT system utilizing digital image correlation (DIC) and a single 2D-calibrated camera was employed to capture deformation during testing. A virtual extensometer with an initial gauge length of 4 mm was used. The recorded force–displacement data provided the basis for evaluating various stress and deformation characteristics, including yield strength (YS), ultimate tensile strength (UTS), uniform elongation (El), and reduction of area (RA).

## 3. Results and Discussion

### 3.1. Additive Manufacturing of 1-MoNiCr Alloy

#### 3.1.1. Analysis of Atomized Powder of 1-MoNiCr Alloy

**Figure 3:**
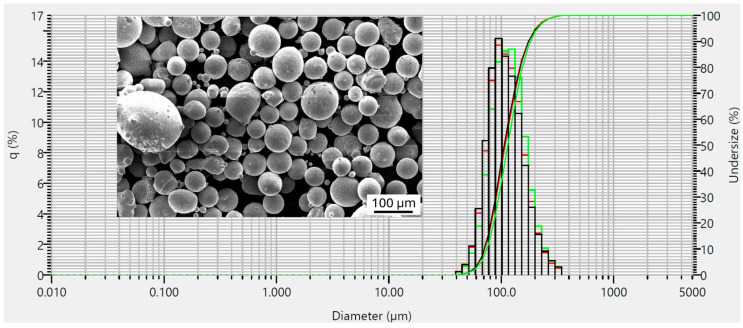
Results of PSD measurements on 1-MoNiCr powder and metallography of 1-MoNiCr alloy powder.

The atomized powder of the alloy 1-MoNiCr was supplied in the 50–200 µm fraction. Therefore, it was first sieved to the desired fraction of 50–150 µm. The powder was measured using the flowability test according to ASTM B213 [24], yielding a result of 15.7 s. Additionally, the powder’s PSD was measured on a HORIBA LA-960 device using the dry method. Three measurements were performed in total, with similar results. The PSD measurement results for the powder 1-MoNiCr (Figure 3) were in the quantiles of d10 = 71 µm, d50 = 109 µm, and d90 = 176 µm. The green, red, and black lines correspond to results obtained from three individual measurements, where each color represents one measurement. The image shows the alloy particles as approximately spherical, globular particles of various sizes. Some particles display an irregular shape or feature smaller attached satellite particles.

#### 3.1.2. Additive Manufacturing of First-Generation SDM800 Module of 1-MoNiCr Alloy

The first phase of deposition was carried out on the first-generation SDM800 module. Cubes of 20 × 20 mm were deposited in various configurations. A constant power of 700 W/350 W was maintained throughout the printing process, with argon cooling, platform preheating to 500 °C, and pause optimization of 3.5 s/10 s between layers or within the contour/fill. The most used mode is DMT, in which the printer automatically adjusts the laser power. In this case, an additional idle laser pass was applied, where the laser passes over the printed layer without powder after the layer is completed.

**Figure 4:**
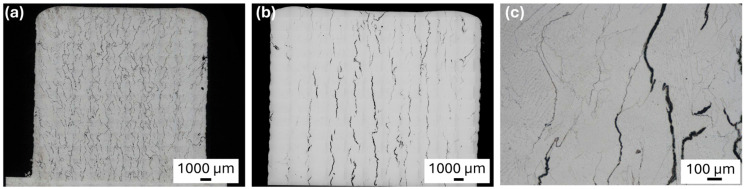
Microstructure (**a**) 800_1-MoNiCr overview image DMT; (**b**) 1600_1-MoNiCr overview image; (**c**) detailed microstructure with the presence of solidification cracks 800_1-MoNiCr.

However, in all the tested printing variants with the 1-MoNiCr material, numerous cracks were detected. Figure 4a shows the microstructure of a sample printed in DMT mode.

In the microstructure of the analyzed samples, solidification cracks were observed (Figure 4c), which are characteristic of nickel-based alloys [12]. These cracks were primarily located along grain boundaries and exhibited a dendritic structure, typical of this type of defect that occurs during the final stages of solidification. This phenomenon is caused by the uneven distribution of impurities in the last phase of solidification, resulting in the localization of residual liquid material at the grain boundaries. During cooling, deformation and stress concentration occur at these boundaries, promoting cavity formation and subsequent crack propagation.

Like other nickel superalloys, these solidification cracks result from a complex interaction of stress and microstructural arrangement during material solidification. The observed cracks are predominantly intergranular, consistent with previously described solidification cracking mechanisms, in which the final portions of the melt solidify along grain boundaries, creating weak spots prone to cracking.

This type of cracking confirms the high susceptibility of the alloy to solidification cracking, which is a common phenomenon in nickel alloys, especially during welding or other rapid solidification processes [12]. The observed cracks emphasize the need to optimize process parameters and investigate additional methods to minimize these defects, ensuring adequate material integrity and mechanical resilience in the final product.

**Figure 5:**
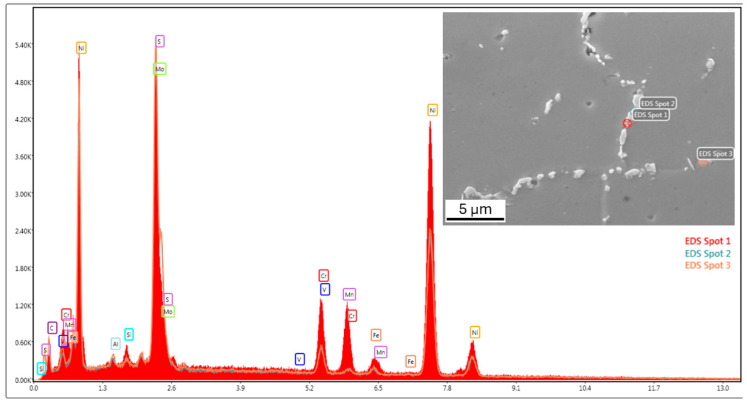
Results of EDS analysis of first-generation sample 800_1-MoNiCr.

Using EDS analysis, elongated particles were observed along grain boundaries in the microstructure (Figure 5), as well as small particles distributed throughout the material. The colors of the curves correspond to the respective EDS spots indicated in the legend (EDS Spots 1, 2, and 3). In the upper section of the print, small, nearly equiaxed grains were present, while crack propagation occurred along the boundaries of columnar grains. As manufactured, this material was unsuitable even for testing mechanical properties.

#### 3.1.3. Additive Manufacturing of Second-Generation SDM1600 Module of 1-MoNiCr Alloy

The second phase of the experiment involved using the SDM1600 module for the deposition of the 1-MoNiCr alloy. The optical systems are similar to those in the first-generation SDM800 module, with differences in the cooling system, powder feed, etc. In this case, two samples were deposited, each in the form of a 20 mm cube. The first sample was deposited using DMT parameters in the range of 750–1050 W, with most of the printing between 900 and 1000 W. The second sample was deposited with a power parameter of 980–1250 W. These two different deposition parameters were chosen based on previous tests and the potential influence of laser power on the result, with the occurrence of cracks being the primary evaluation criterion.

The results exhibited a similar trend as those observed in the first phase of the experiment with the 1-MoNiCr alloy (Figure 4b), indicating persistent issues with the formation of solidification cracks. These cracks appeared despite the use of different laser power parameters and the technical improvements implemented in the SDM1600 module. Although varied laser power settings (750–1050 W for the first sample and 980–1250 W for the second sample) were selected to potentially influence the solidification process and reduce the likelihood of cracking, the alloy continued to display cohesion issues associated with solidification defects.

Solidification cracks typically arise during the final stages of solidification, where an uneven distribution of solutes along grain boundaries creates weak points. These weak zones become susceptible to crack formation due to localized residual stress. The findings indicate that, despite optimized deposition parameters and an improved cooling system in the SDM1600 module, no significant reduction in crack formation was achieved. This suggests that adjusting only the laser power parameters and technical enhancements may be insufficient to fully eliminate problematic solidification defects in the 1-MoNiCr alloy. Consequently, these results emphasize the need for further process adjustments and potential modifications in alloy composition to enhance material cohesion and minimize the occurrence of undesirable cracks.

#### 3.1.4. Analysis of Impurities in 1-MoNiCr Alloy

**Figure 6:**
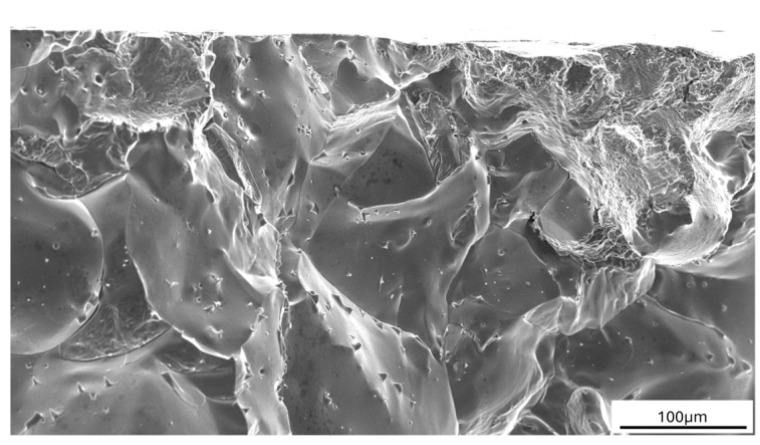
Fracture surface of 1-MoNiCr alloy after tensile test.

**Figure 7:**
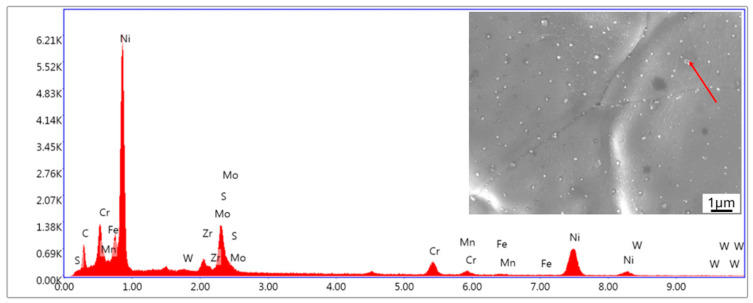
Results of EDS analysis of 1-MoNiCr alloy (red arrow—sulfur).

Several variants of print parameter settings were tested; however, a suitable material free of cracks was not achieved. For this reason, attention was redirected toward optimizing the chemical composition of the alloy, as described in Section 2.1. The goal was to improve material purity, with a particular focus on reducing the sulfur, oxygen, and nitrogen content. Figure 6 shows an intergranular fracture from a tensile test on conventionally produced 1-MoNiCr material, i.e., cast and forged, where sulfur was detected along the grain boundaries (Figure 7), which subsequently also contributed to crack formation during additive manufacturing.

A detailed analysis revealed that material decohesion occurred due to a combination of two main factors: the presence of sulfur and the occurrence of solidification cracks. Sulfur, known for its detrimental effect on grain boundary cohesion, accumulated along grain boundaries, creating weakened areas. These weakened zones became susceptible to the formation of solidification cracks, which propagated along the grain boundaries and significantly compromised the structural integrity of the material.

As a result, the material produced in this manner was insufficiently cohesive and did not meet the quality standards for further processing. Consequently, it was not suitable for mechanical property testing, as its compromised structure would yield results that were not representative of its performance in practical applications. These findings underscore the importance of controlling impurities and optimizing process parameters to achieve materials with high structural and mechanical stability.

### 3.2. Additive Manufacturing of 2-MoNiCr Alloy

As mentioned above, due to the unsuccessful additive manufacturing of the 1-MoNiCr material, the chemical composition of the MoNiCr alloy was optimized to address the identified issues. The results of this study align with findings from Hu et al. [29], who examined the mechanism of intergranular embrittlement caused by sulfur and its effect on the embrittlement of nickel alloys. By reducing the concentrations of sulfur and other impurities, the material’s purity can be improved, leading to enhanced grain cohesion and increased resistance to crack formation. The significance of reducing these elements, as demonstrated in the chemical analysis (Table 2), lies in its positive impact on structural integrity and suitability for additive manufacturing applications.

#### 3.2.1. Analysis of Atomized Powder of 2-MoNiCr Alloy

**Figure 8:**
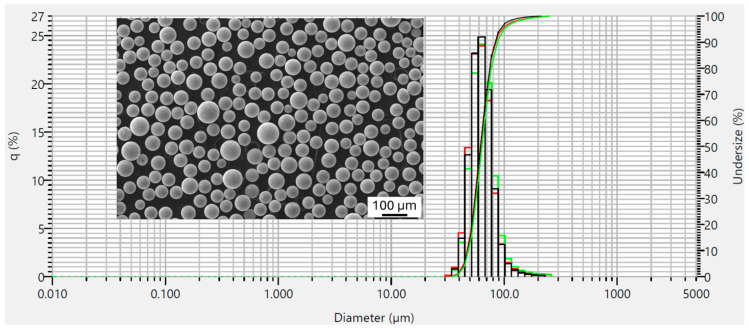
Results of PSD measurements on 2-MoNiCr powder and metallography of 2-MoNiCr alloy powder.

The conventionally produced material with optimized chemistry, the 2-MoNiCr alloy (Table 2), was atomized into powder with a fraction size of 50–125 µm. The powder was tested for flowability according to ASTM B213 [24], resulting in 11.6 s. Additionally, the PSD was measured on a HORIBA LA-960 device using the dry method. Three measurements were conducted in total, yielding similar results. The PSD measurement results for the 2-MoNiCr powder (Figure 8) showed quantiles of d10 = 47 µm, d50 = 62 µm, and d90 = 85 µm. The green, red, and black lines correspond to results obtained from three individual measurements, with each color representing one measurement. The powder is spherical and free from unwanted satellites.

Overall, the 2-MoNiCr alloy powder demonstrates better flowability and a narrower particle size distribution than the 1-MoNiCr alloy powder. Additionally, the 2-MoNiCr powder exhibits a more consistent particle shape, which may positively impact its properties in the additive manufacturing process, where high flowability and uniform powder distribution are required. The 1-MoNiCr alloy powder has a broader size range and the presence of satellites, which may impair its flowability and uniformity during layer deposition. The 2-MoNiCr powder demonstrated superior flowability and a more consistent Gaussian particle size distribution compared to the 1-MoNiCr powder.

Studies [21,22,30] suggest that the characteristics of powder, particularly particle size distribution and shape uniformity, significantly impact flowability and deposition uniformity, which are critical in achieving high-quality deposited parts. The 2-MoNiCr alloy powder, with better flowability and a narrower PSD, aligns well with findings indicating that more consistent particle shapes and narrower PSDs lead to improved layer uniformity during the additive manufacturing process [22]. In contrast, the 1-MoNiCr powder, with a broader size range and satellite particles, may experience reduced flowability. The presence of satellites and irregularly shaped particles in 1-MoNiCr could further hinder the deposition quality, emphasizing the importance of optimized powder characteristics for achieving superior mechanical and structural properties in additively manufactured components.

#### 3.2.2. Additive Manufacturing of Second-Generation SDM1600 Module of 2-MoNiCr Alloy

The optimized 2-MoNiCr alloy was tested exclusively on the SDM1600 device, as this module allows for a higher deposition rate and is better suited to the requirements of industrial applications, thereby enhancing process efficiency and the quality of the final material. Similarly to the deposition of the 1-MoNiCr powder, two samples, each in the form of a 20 mm cube, were deposited using the SDM1600 module (Figure 1). The first sample was deposited under DMT parameters in the range of 750–1050 W, with most of the printing between 900 and 1000 W. The second sample was deposited with a power parameter of 980–1250 W.

**Figure 9:**
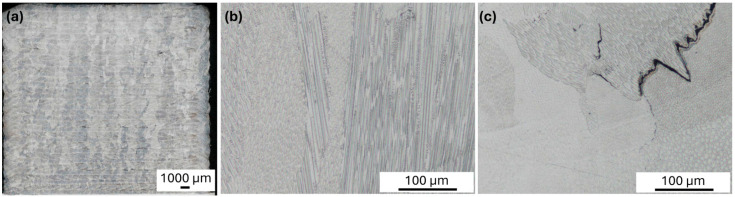
Alloy 1600_2-MoNiCr: (**a**) overview of the sample after additive manufacturing; (**b**) metallographic analysis of the sample after additive manufacturing; (**c**) solidification crack after additive manufacturing.

The microstructure of the additively manufactured material, as shown in Figure 9a–c, exhibits several distinctive features. The microstructure reflects the unique characteristics of additive manufacturing, including directional grain growth, a layer-by-layer structure, and potential defects such as solidification cracks, which impact the material’s mechanical properties. The overview image (Figure 9a) displays the layered structure typical of additive manufacturing, with visible layer boundaries resulting from the sequential deposition process. Each layer reflects the deposition path, indicating the formation of melt pools during the process. A closer view (Figure 9b) reveals a columnar grain structure aligned along the build direction, a common feature in additively manufactured materials. These elongated grains are indicative of the rapid cooling and directional solidification that occur during the laser-based deposition process. The metallographic analysis of the samples produced from the optimized 2-MoNiCr alloy powder revealed only a minimal amount of solidification cracks. Figure 9c shows solidification cracks located along grain boundaries, a common defect in nickel-based alloys produced by additive manufacturing. The cracks appear intergranular, likely due to thermal stresses and solute segregation during the final stages of solidification. This observation underscores the need for the careful control of processing parameters to minimize cracking and improve structural integrity.

Solidification cracking in MoNiCr was primarily mitigated through careful alloy optimization, particularly by minimizing impurities such as sulfur, phosphorus, and oxygen. Studies on nickel-based alloys highlight that sulfur and phosphorus tend to segregate at grain boundaries, leading to embrittlement and the formation of low-melting-point phases that promote cracking during the final stages of solidification [12]. By reducing these impurities, the MoNiCr alloy demonstrated improved grain boundary cohesion and resistance to crack initiation. This indicates that the adjustments made to the chemical composition were effective in reducing the formation of these defects, which are typically a challenge in nickel-based alloys [21]. The optimized composition appears to have improved the solidification process, resulting in a more uniform and defect-resistant microstructure. This reduction in solidification cracking enhances the structural integrity and overall performance of the material, making it better suited for applications requiring high mechanical strength and durability.

In addition to reducing the levels of typical harmful elements (S, O, and P), it is also essential to achieve a reduction in the content of other associated elements such as Al, Ti, Co, Cr, Mn, Cu, and Si, as well as C and N. These elements can either directly contribute to the formation of unwanted phases or increase the reactivity of other elements by their presence. The listed elements can form uniformly distributed precipitates—typically Ni_3_(Al,Ti)—or minor phases with preferential occurrences. For instance, Ni, Cr, Mo, and Co are elements that commonly form TPC-type (Topological Close-Packed) phases, which can attack grain boundaries with detrimental effects [31,32]. Although the reduction in the content of individual elements may seem relatively minor, the cumulative effect can have a significant impact on the material’s properties. MoNiCr is intended as a single-phase material, and the formation of any minor phases is undesirable. Therefore, reducing the levels of these associated elements can contribute to improved technological and functional properties.

The chemical composition of the MoNiCr alloy was tailored to address challenges in additive manufacturing, particularly by reducing elements such as sulfur, phosphorus, and oxygen, which are known to promote cracking and embrittlement. Compared to standard formulations like Hastelloy-N^®^ and GH3535^®^, MoNiCr features a significantly lower content of these impurities, as well as reduced levels of less noble elements like manganese, silicon, and chromium, which tend to segregate at grain boundaries and form low-melting phases [1]. These adjustments aim to enhance the alloy’s grain boundary cohesion and mitigate the formation of detrimental second-phase carbides, such as M_6_C, which can weaken the material and increase susceptibility to cracking [1]. The higher nickel and molybdenum content in MoNiCr also contributes to its improved resistance to solidification defects and mechanical performance under high-stress conditions, distinguishing it from conventional alloys used in high-temperature applications.

#### 3.2.3. Heat Treatment of 2-MoNiCr Alloy

**Figure 10:**
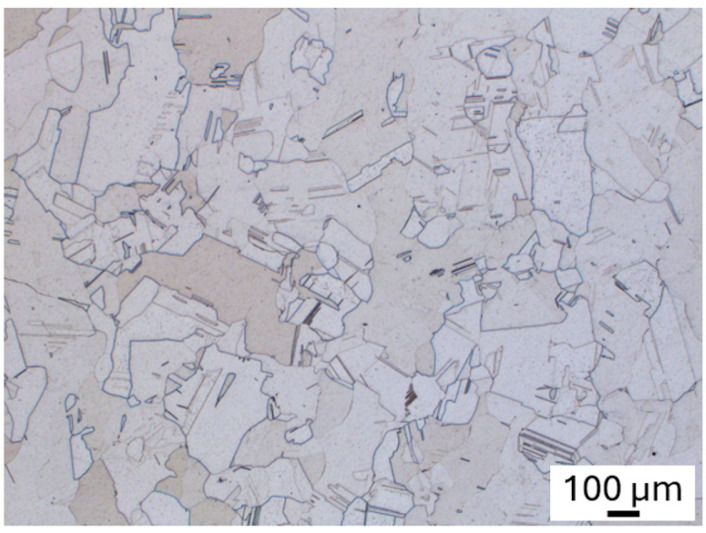
Metallographic analysis of the sample 1600_SA_2-MoNiCr after additive manufacturing and heat treatment.

Post-processing heat treatments were applied to reduce residual stresses and homogenize the microstructure, improving the isotropy of the material. These strategies effectively enhanced the material’s structural integrity and mechanical properties. The process involved solution annealing at 1200 °C followed by rapid cooling. The microstructure observed after this heat treatment displayed finer, equiaxed grains with no visible traces of the initial melt pools resulting from the prior additive process. This change in grain structure and the removal of melt pool remnants indicate that the heat treatment was successful in enhancing the uniformity of the microstructure, leading to improved material properties suitable for demanding applications (Figure 10).

**Figure 11:**
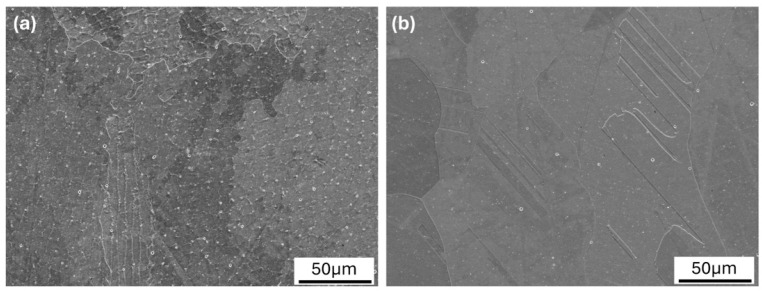
SEM Micrographs of 2-MoNiCr Alloy in the (**a**) 1600_2-MoNiCr, (**b**) 1600_SA_2-MoNiCr.

The detailed SEM analysis revealed minimal defects in the microstructure of the 2-MoNiCr alloy, with very low porosity and well-defined grain structures. The microstructural analysis of the additively manufactured samples was conducted using SEM for both the as-built (1600_2-MoNiCr) and solution-annealed (1600_SA_2-MoNiCr) states. The SEM micrographs (Figure 11a,b) reveal distinctive features in the microstructures. 

The presence of cracks in the material was quantified by determining the area fraction of cracks within the analyzed microstructure (Table 6). This assessment was performed using NIS Elements 5.2 digital image processing and analysis software, which enabled precise quantification of the cracked regions. This analysis ensures an accurate and consistent evaluation of the material’s integrity.

#### 3.2.4. Mechanical Properties of 2-MoNiCr Alloy

Subsequently, the influence of the microstructure on the resulting mechanical properties was examined. This analysis aimed to understand how the refined, equiaxed grain structure, achieved through heat treatment, affected the material’s strength, ductility, and overall performance.

**Figure 12:**
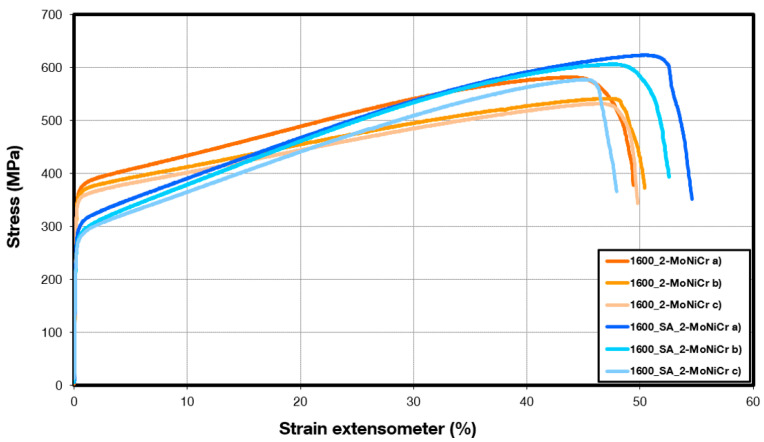
Results from tensile tests after additive manufacturing and heat treatment of 2-MoNiCr alloy.

Table 7 and Figure 12 present the results of the tensile test of the 2-MoNiCr alloy. The properties were measured on the sample in both the as-built condition, directly after the additive manufacturing process (1600_2-MoNiCr), and after subsequent solution annealing using miniaturized tensile tests-MTT (1600_SA_2-MoNiCr).

The mechanical property results show differences between the various material states. The highest yield strength (YS) is achieved by the as-built samples (1600_2-MoNiCr), with an average value of 356 ± 7 MPa, accompanied by a moderate scatter in the ultimate tensile strength (UTS), which averages 551 ± 21 MPa. The solution-annealed state (1600_SA_2-MoNiCr) exhibits a slightly lower yield strength, reduced to 279 ± 10 MPa, but demonstrates higher UTS values compared to the as-built state, averaging 602 ± 19 MPa.

The state after additive manufacturing without solution annealing corresponds to a cast structure, where dendrites and associated segregation are still visible. In contrast, the state after additive manufacturing and solution annealing exhibits a recrystallized finer structure typical of an FCC solid solution in nickel alloys, with numerous annealing twins. The higher yield strength observed in the non-annealed deposited state could be explained by the presence of strengthening particles of intermediate phases. These phases were eliminated through solution annealing, resulting in a lower yield strength in the annealed state. However, the annealed state, free from intermediate phases, exhibits a greater potential for work hardening, resulting in a higher ultimate tensile strength during tensile testing.

The observed reduction in defects (Figure 9, Figure 10 and Figure 11) and the heat treatment directly correlate with enhanced mechanical properties, including a yield strength of 279 MPa, an ultimate tensile strength of 602 MPa, and elongation at break of 51%. These results demonstrate the effectiveness of the alloy’s composition, microstructural homogeneity, and processing methods. As emphasized in [33], the selection of the process depends on the material system, the desired properties, and the application requirements. These findings underscore the suitability of the optimized 2-MoNiCr alloy for high-performance applications.

The mechanical properties observed in the Hastelloy N alloy [34] exhibit higher tensile strength (approximately 760 MPa) and good yield strength (290 MPa in the base metal and 380 MPa in the welded joints). The lower mechanical properties of additively manufactured MoNiCr can be contrasted with conventionally produced Hastelloy N, which was fabricated through a conventional manufacturing method, which imparts a “material history” of deformation, resulting in a refined grain structure and that carries through even into the weld metal. These characteristics contribute to Hastelloy N’s strength and ductility, leveraging the inherent benefits of the rolling process, such as grain refinement and work hardening. In contrast, additively manufactured MoNiCr lacks this deformation history.

Challenges such as anisotropy, porosity, and residual stresses were addressed through the optimization of process parameters, including laser power, scanning speed, and layer thickness, to ensure uniform material deposition and improve structural integrity. Additionally, exploring post-processing techniques, such as heat treatment and hot isostatic pressing (HIP), can further enhance mechanical properties and structural integrity.

Additive manufacturing demonstrates clear superiority for the MoNiCr alloy by enabling advanced capabilities, such as the creation of FGMs with tailored properties that cannot be achieved through traditional methods. The PBF-LB process offers unmatched precision and the ability to produce fine microstructures, while the hybrid combination of DED-LB and PBF-LB enables scalable manufacturing with localized property control. These unique advantages make AM the superior choice for producing MoNiCr components, particularly for applications in demanding environments where conventional methods fall short.

The current research conducted on dimensionally limited samples will be transferable to larger pieces, including industrially produced components for energy devices. Semi-finished products weighing several kilograms can already be prepared through melting, casting, forming, and heat treatment. Scaling up to even larger components will depend on the effective use of predictive numerical calculations and their correct interpretation to determine technological parameters. In additive manufacturing, it is necessary to respect key parameters such as deposition speed, heat removal conditions, and the quality of the powder used in the production of large components to avoid exceeding the specified temperature gradient, internal stresses in the deposited part, segregation, or even the nucleation of solidification cracks.

## 4. Conclusions

Research successfully demonstrated the potential of the MoNiCr alloy for additive manufacturing in advanced energy applications, particularly through the optimization of its chemical composition and processing parameters. The initially tested 1-MoNiCr alloy exhibited issues with solidification cracking, which led to intergranular cracks that compromised the material’s structural integrity. By refining the chemical composition to reduce impurities such as sulfur, phosphorus, and oxygen, the development of the 2-MoNiCr alloy achieved a significant reduction in defect formation, resulting in a more cohesive and defect-resistant microstructure.

Mechanical property tests confirmed that the as-built 2-MoNiCr alloy demonstrated a yield strength of 356 ± 7 MPa and an ultimate tensile strength of 551 ± 21 MPa. The post-processing heat treatment further improved its tensile properties, with the solution-annealed samples achieving an ultimate tensile strength of 602 ± 19 MPa, while the yield strength slightly decreased to 279 ± 10 MPa. The microstructure after solution annealing revealed the typical appearance of an FCC solid solution in the nickel alloys following the deformation and recrystallization process. The fine, equiaxed grains contained numerous annealing twins, which are characteristic of FCC metals after deformation and annealing.

Future research will focus on corrosion testing of the developed MoNiCr alloy to evaluate its performance in demanding environments. Additionally, efforts will explore other additive manufacturing processes, such as wire DED-LB and PBF-LB, along with post-processing techniques like heat treatment and HIP, to further expand processing possibilities and optimize the alloy for various high-performance applications in the energy sector.

## Figures and Tables

**Table 1:** Chemical composition of 1-MoNiCr alloy, wt. %.

C	Si	Mn	P	S	Cr	Fe	Mo	Cu	Al	Ti	V	W	Nb	Co	N	O	Ni
0.02	0.25	0.49	0.009	0.012	6.72	2.0	15.7	0.009	0.26	0.04	<0.022	0.082	0.008	0.05	0.0079	0.0062	balance

**Table 2:** Chemical composition of 2-MoNiCr alloy, wt. %.

C	Si	Mn	P	S	Cr	Fe	Mo	Cu	Al	Ti	V	W	Nb	B	N	O	Ni
<0.001	0.19	0.18	<0.003	<0.003	5.8	2.3	16.88	0.002	0.23	0.008	0.019	0.08	0.02	0.001	0.0025	0.0012	balance

**Table 3:** Printing parameters for module SDM800.

Laser spot size (µm)	800
Layer height (µm)	250
Hatch distance (µm)	500
Travel speed (mm/min)	850
Powder amount (g/min)	3
Protective gases (coaxial + shield + powder) (L/min)	13
Range of laser power for DMT mode (W)	300–650
Printing strategy	ZigZag CFC + CF

**Table 4:** Printing parameters for module SDM1600.

Laser spot size (µm)	1600
Layer height (µm)	600
Hatch distance (µm)	1100
Travel speed [mm/min]	850
Powder amount (g/min)	5.6
Protective gases (coaxial + shield + powder) (L/min)	23
Range of laser power for DMT mode (W)	750–1050 and 980–1250
Printing strategy	ZigZag CFC + CF

**Table 5:** Overview and labeling of additively manufactured (AM) samples.

Sample	Processing Status
800_1-MoNiCr	AM with module SDM800 of 1-MoNiCr alloy
1600_1-MoNiCr	AM with module SDM1600 of 1-MoNiCr alloy
1600_2-MoNiCr	AM with module SDM1600 of 1-MoNiCr alloy
1600_SA_2-MoNiCr	AM with module SDM1600 and solution annealing of 1-MoNiCr alloy

**Table 6:** Quantitative analysis of defects of 2-MoNiCr Alloy in as-built and solution-annealed states.

Sample	800_1-MoNiCr	1600_1-MoNiCr	1600_2-MoNiCr	1600_SA_2-MoNiCr
Defect Area Fraction (%)	2.58	2.75	0.03	0.02

**Table 7:** Results from tensile tests after additive manufacturing and solution annealing of 2-MoNiCr alloy.

Specimen	YS	UTS	El	RA
	MPa	MPa	%	%
1600_2-MoNiCr a	364	581	49.2	75
1600_2-MoNiCr b	357	541	50.2	67
1600_2-MoNiCr c	348	532	49.6	73
Average	356 ± 7	551 ± 21	50 ± 0	72 ± 3
1600_SA_2-MoNiCr a	292	623	54.4	66
1600_SA_2-MoNiCr b	274	606	52.3	73
1600_SA_2-MoNiCr c	270	577	47.7	63
Average	279 ± 10	602 ± 19	51 ± 3	67 ± 4

## Data Availability

The original contributions presented in the study are included in the article; further inquiries can be directed at the corresponding author.
